# Molecular Basis and Current Treatment for Alcoholic Liver Disease

**DOI:** 10.3390/ijerph7051872

**Published:** 2010-04-27

**Authors:** Alejandra Miranda-Mendez, Alejandro Lugo-Baruqui, Juan Armendariz-Borunda

**Affiliations:** 1 Institute for Molecular Biology in Medicine and Gene Therapy, University of Guadalajara, Jalisco 44281, Mexico; E-Mails: alemirme@hotmail.com (A.M.M.); alecslugo@yahoo.com (A.L.B.); 2 OPD Hospital Civil de Guadalajara, Jalisco 44340, Mexico

**Keywords:** alcohol, alcoholism, fibrosis, cirrhosis, alcoholic liver disease

## Abstract

Alcohol use disorders and alcohol dependency affect millions of individuals worldwide. The impact of these facts lies in the elevated social and economic costs. Alcoholic liver disease is caused by acute and chronic exposure to ethanol which promotes oxidative stress and inflammatory response. Chronic consumption of ethanol implies liver steatosis, which is the first morphological change in the liver, followed by liver fibrosis and cirrhosis. This review comprises a broad approach of alcohol use disorders, and a more specific assessment of the pathophysiologic molecular basis, and genetics, as well as clinical presentation and current modalities of treatment for alcoholic liver disease.

## 1. Introduction

Alcohol dependency (AD) is a complex addictive disorder, affecting millions of individuals around the world each year. AD is considered a worldwide public health problem with a direct causal relationship between alcohol consumption and more than 60 different types of disease and injury. Regarding this, the ICD-10 (International Classification of Disease 10th Revision) has reported eight causes of death related to alcohol consumption (Table 1). Most of these causes are related to the amount and time of exposure to alcoholic beverages. According to the World Health Organization, the average alcohol consumption per capita in adults is 5.1 liters per year, though mean consumption varies worldwide. While in some countries, like Saudi Arabia, alcohol consumption (liters per capita per year) is 0.0, in other countries such as Luxembourg the consumption reaches 17.54 [1].

The impact and consequences of AD and alcohol abuse have elevated economical costs, as well as negative health and social implications. The most direct health influence of ethanol consumption is reflected in a systemic level; thereby moderate ethanol consumption has been related to a reduced cardiovascular disease, favorable lipid profile and less thrombogenic platelet function [2]. On the other hand, elevated amounts of alcohol intake lead to hepatic damage.

Although the mechanisms by which ethanol causes liver damage have been well described, some facts remain to be elucidated. The risk, severity and prognosis of ethanol-mediated liver diseases generally depend on the amount, frequency, and duration of alcohol consumption, as well as the continued presence of liver inflammation, diet, nutritional status and genetic make-up of the host. In this matter, alcoholic steatosis (AS) is the initial pathology found in early stages of the natural history of alcoholic liver disease (ALD). AS is characterized by the accumulation of lipids in the liver. Steatosis caused by alcohol is clinically considered as benign since it is reversible if the stimulus is removed. The progression to alcoholic steatohepatitis (ASH) represents the key step in the development of ALD. Only a small portion of heavy drinkers develop a severe form of the disease, suggesting the involvement of other factors modifying ALD onset and presentation [3].

Alcohol is identified as a harmful agent to the liver, and hepatic stellate cells (HSC) are activated in response to this injury. Activated HSC are recognized as fibrogenic cells and lead to deposition of collagen (particularly collagen type I). HSC are also regulated in a paracrine manner by acetaldehyde and reactive oxygen species (ROS), produced by the metabolism of ethanol in surrounding hepatocytes. Activated Kupffer cells secrete pro-inflammatory cytokines, linking apoptosis in the liver to inflammation [4].

“Programmed cell death”, also called apoptosis, plays an important role in the pathogenesis of chronic liver disease. Whenever there is an acute presentation of the disease, it is possible to observe massive hepatocyte apoptosis. This phenomenon induces progressive fibrosis, which is the hallmark of chronic liver damage and, therefore could result in liver failure, cirrhosis, and hepatocellular carcinoma.

To objectively assess damage degree in the hepatic parenchyma, completion of a histological study is required. In steatohepatitis, findings in the lamellae include hepatocyte steatosis, ballooning, apoptosis, and lobular inflammatory infiltrate [4]. When ALD is established, an accumulation of reducing equivalents in the cytosol and the rates of fatty acids biosynthesis and subsequent esterification into triglycerols are markedly increased [5].

## 2. Assessing the Problem

The World Health Organization reports about two billion alcohol consummers worldwide and 76.3 million people with diagnosable alcohol use disorders [1]. Globally, alcohol causes 3.2% deaths for all causes (1.8 million deaths annually) and accounts for 4.0% of disease burden [2].

Excessive ethanol consumption is an important preventable cause of morbidity. The association between ethanol intake and alcoholic liver disease has been well documented, though liver cirrhosis develops only in a small proportion of heavy drinkers. Twenty percent of alcoholics and heavy drinkers develop fatty liver and only 10 to 15 percent of these will develop cirrhosis. The point prevalence of cirrhosis is 1% in people who drink 30 to 60 g of alcohol a day, and up to 5.7% in those consuming 120 g daily. In this matter, mortality studies have demonstrated that heavy drinkers and alcoholics die from cirrhosis at a much higher rate than the general population [6].

A clear aggregated environmental risk factor for the development of cirrhosis attributable to alcohol consumption is infection with hepatitis C virus (HCV). The risk of cirrhosis in humans infected with HCV increases proportionally with consumption of more than 30 g of alcohol per day; as mentioned above, the highest risk is associated with consumption of more than 120 g per day. Other factors, such as sex, genetic characteristics, and environmental influences (including chronic viral infection), play a role in the genesis of ALD [7].

In addition, alcoholism is a multifactorial brain disorder, according to the DSMIV (Diagnostic and Statistical Manual of Mental Disorders Fourth Revision), therefore the approach should be addressed on neurobiological and genetic strategies for the prevention, diagnosis, and treatment of alcoholism. The identified factors that contribute to the development of alcoholism are included in a social environment (*i.e.*, parents, family, friends, peers, community, society). Possible social co-determinants such as poverty, social inequality, and easy availability of alcoholic beverages might establish alcoholism development.

Several studies indicate that AD has a genetic component. The heritability of alcohol dependence is rather high, ranging between 50% and 60%, though it has been shown to be a complex polygenic disorder. There have been associated specific genes and polymorphisms that affect the risk of alcohol use disorders. These genes involve expression of enzymes responsible for alcohol metabolism, such as alcohol dehydrogenase and aldehyde dehydrogenase; those associated with disinhibition, and those that confer a low sensitivity to alcohol [8].

## 3. Genetic Predisposition

Genes have been proposed to be involved in a predisposition to ALD development and alcohol use disorders. Combined genetic information about these two entities might enhance alcohol related diseases.

### 3.1. Candidate Genes Predisposing Alcoholic Liver Disease

When ethanol is consumed, its metabolism depends almost exclusively on the liver through different enzymes capable of catalyzing the oxidation of alcohol. Polymorphisms in the two main enzymes involved in ethanol metabolism [alcohol dehydrogenase; 1B (ADH1B) and aldehyde dehydrogenase (ALDH2)] confer susceptibility to develop ALD. Alcohol is transformed into acetaldehyde through alcohol dehydrogenases, this intermediate metabolite is responsible for the toxic effects of ethanol. The next step is the metabolism of acetaldehyde to acetate (a non-toxic metabolite) through ALDH2 [9]. Either higher activity of ADH1B or lower activity of ALDH2 lead to accumulation of acetaldehyde following alcohol consumption, which, in turn, causes an unpleasant and aversive response, termed the flushing reaction, similar to the disulfiram effect [10]. Japanese, Chinese and some Jewish population constitute the main group displaying specific protective polymorphisms against the development of ALD [11].

### 3.2. Candidate Genes Predisposing Alcohol Use Disorders

Linkage studies have identified several chromosomal regions, supporting the involvement of loci on chromosomes in alcohol dependency, however the recognition of specific gene regions is still challenging.

#### 3.2.1. Endophenotypes

It is possible that a small number of genes might directly influence alcohol dependency. Nevertheless, it is more likely that relevant genes affect a range of endophenotypes (defined as genetically influenced intermediate characteristics). These endophenotypes will subsequently have impact in the risk for heavier drinking and alcohol-related life problems. Some of these genetically influenced characteristics are related to alcoholism, focused in treatment response [12]. In contrast to what happens with protective polymorphism in ALD, a low response to alcohol predicts increased risk of developing alcohol use disorders and has been associated with genetic variation in the serotonin transporter gene and the gene encoding γ-aminobutyric acid receptor A (GABRA) [11].

It has also been suggested that a determined gene—environmental interaction contributes to the development of AD. This occurs when exposure to an environmental factor has an effect on the health of individuals which is conditioned to their genotype. Thus, several genes have been described including monoamine oxidase A (*MAOA*), the serotonin transporter (*HTT*), catechol-O-methyltransferase (*COMT*), the corticotropin releasing hormones receptor 1 gene and the dopamine transporter [8,11,13–15]. The D2 dopamine receptor gene is the most widely studied and described receptor related to alcoholism [16]. One study showed that the prevalence of the *DRD2* minor allele A1 (A1/A1 or A1/A2 genotype) was significantly higher in alcoholics than in nonalcoholic controls (*p* = 0.001). Mutations in two regions of the DRD2 gene (*Taq*I A and *Taq*I B), are associated with alcoholism and other drug use disorders [13]. Regarding this matter, a RFLP in the TaqI A1 allele is strongly associated with severe alcoholism. Another study demonstrated that the frequency of the A1 allele at DRD2 in descendants of alcoholic parents was significantly higher than in children of non alcoholics. It was also shown a significant association between the genotype at DRD4 and the children of alcoholic patients [16]. Although there is a wide range of candidate genes related to alcoholism, further studies are needed, not only to elucidate the genetic etiology of this entity, but to decipher therapeutic targets.

## 4. Pathophysiology

Mechanisms implicated in alcohol-induced liver damage involve many biochemical reactions with different simultaneous pathways interacting with each other. These mechanisms involve enzymes, ROS, endotoxins, cytokines, immune system cells, and genetic predisposition to develop alcoholic liver disease [17,18]. Alcohol is metabolized by oxdative reactions to acetaldehyde and then converted to acetate through alcohol dehydrogenase (ADH) and acetaldehyde dehydrogenase (ALDH). ADH is quantitatively the most important enzyme capable of catalysing the conversion of ethanol to acetaldehyde.

**Figure 1.**
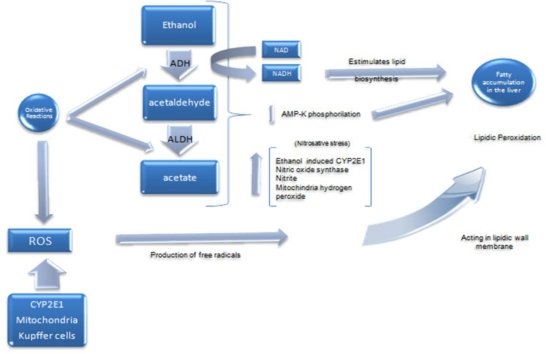
Main molecular mechanisms by which ethanol causes oxidative stress. NAD Nicotinamide adenine dinucleotide, NADH reduced form of NAD, AMP-K Adenosine monophosphate protein kinase, ROS Reactive oxygen species, ADH Alcohol dehydrogenase, ALDH Acetaldehyde dehydrogenase.

It has been proposed that ethanol metabolism causes proteasome inhibition [19] and that the initial molecular changes of ethanol injury are the reduction of both phosphorylation and activity of AMP-dependent protein kinase (AMPK). This contributes to the development of fatty liver by allowing increased activity of acetyl-CoA carboxylase [20,21]. Although alcoholic fatty liver resolves with alcohol abstinence, steatosis predisposes to the development of hepatic fibrosis and cirrhosis in people with continued pathological drinking patterns (Figure 1) [6].

Wu *et al*. mention that several mechanisms contribute to alcohol-induced liver injury, and that ethanol-induced oxidative stress is likely to arise from several sources, including CYP2E1, mitochondria, and activated Kupffer cells [17,22]. A central pathway appears to be the induction of cytochrome P450 2E1. It metabolizes and activates many toxicological substrates, including ethanol, to more reactive and toxic products [23]. This mechanism plays a role in creating a harmful condition known as oxidative stress, defined as the result of an imbalance between pro-oxidant and antioxidant systems [20]. This enhances the formation of free radicals which act in the lipidic wall membrane ongoing lipid peroxidation.

ROS are intermediates in cell signaling pathways that alter gene expression and lead to cell proliferation, migration, and apoptosis; thus contribute to the development of chronic ethanol-induced liver injury [23]. When alcohol is consumed, CYP2E1 generates acetaldehyde as well as ROS in the cytosol of the cell, leading to an increase in oxidative stress with a probable mitochondrial origin. ROS are toxic to cells because they can react with most cellular macromolecules by inactivating enzymes or denaturing proteins, which causes DNA damage [6].

When alcohol is metabolized to acetaldehyde, the oxidized form NAD converts to the reduced form NADH. This reaction generates an excess of reduced equivalents which stimulates lipid biosynthesis [24]. Alcohol exposure increases nitrosative stress with elevated levels of ethanol-inducible CYP2E1, nitric oxide synthase, nitrite and mitochondrial hydrogen peroxide. Ethanol can also induce and/or activate inducible nitric oxid synthase (*i*NOS), which plays a key role in worsening alcohol-mediated hepatotoxicity. Moreover, the overexpression of CYP2E1 aggravates hepatic injury. Besides, expression of cytokeratins 8 and 18 can be considered as biomakers for ALD progression [25].

Alcohol intake also increases gut permeability, allowing bacterial endotoxins to enter the bloodstream and liver, where it can activate certain immune cells. Lipopolysaccharide (LPS) is an important activator of Kupffer cells requiring three cell proteins to complete the process: CD14, TLR4, and MD2. Activated Kupffer cells are responsible for an increased expression of TNFα and multiple inflammatory cytokines [26]. TNF-α is a mediator, not only in the early stages of fatty liver disease but also in the transition to more advanced stages of liver damage [27]. This activation is one of the earliest liver responses to injury. TNF-α is considered one of the most important cytokines involved in the pathogenesis of alcoholic liver bruise. Evidence indicates that disruption of the TNF-αR1 gene or treatment with a TNF-α neutralizing antibody, reduces alcoholic liver injury in mice.

**Figure 2.**
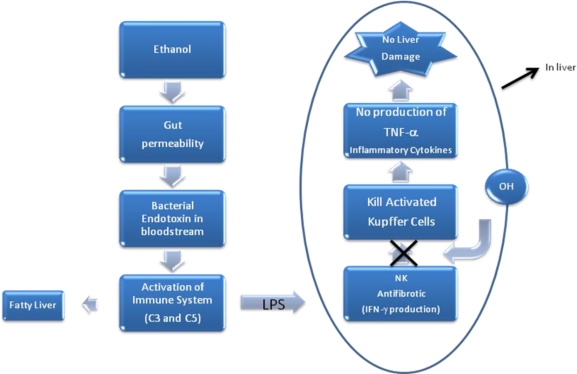
NK Natural killer, TNF-α Tumor Necrosis Factor alpha, LPS Lipopolysaccharide.

Alcohol-induced liver damage also depends on the host immune system susceptibility. Several studies have suggested that immune response plays a role in alcoholic liver disease. Pritchard *et al*. suggested the hypothesis that activation of the complement is required for the development of ethanol-induced fatty liver. They found that C3 contributed to accumulation of triglyceride in the liver, while C5 was involved in inflammation and injury to hepatocytes [28]. Another study demonstrated that NK cells may also directly ameliorate hepatic fibrosis, first by releasing antifibrotic cytokines (interferon [IFN]*α* and IFN*γ*), and second by killing activated stellate cells. Ethanol impairs the ability of NK cells to kill activated HSC. As a result, it could remove this break in the development of fibrosis (Figure 2) [29].

Moreover, there are some underlying conditions in which ethanol can cause damage in a broader manner. Thereby, ethanol and chronic HCV infection synergistically accelerate liver injury. The process by which this occurs remains unclear, but it is believed that chronic liver inflammation is the keyhole to this pathogenesis. In these cases, there is a relationship between increased alcohol intake and decreased response to interferon (IFN) therapy for HCV [7].

Chronic consumption of alcohol elevates iron levels in the body, not only when consuming iron-rich beverages like wine, but also stimulating exogenous iron absorption from food.

In general, cells have protective mechanisms to attenuate harm caused by ROS and the oxidative process. These mechanisms include enzymes like SOD (Superoxide Dismutase), catalase, glutathione peroxidase (which removes H_2_O_2_), glutathione transferase, ferritine (removes metals like iron that promotes oxidative stress), and low molecular weight antioxidants such as vitamin E, vitamin C, vitamin A, uric acid, and bulirubin. However, the production of oxidative species and reduced equivalents is more elevated than defense mechanisms, due to the fact that alcohol inhibits antioxidants [30].

### 4.1. Clinical Presentation of Alcoholic Liver Disease

There is a wide variety of clinical presentations of ALD. Symptoms depend on the severity and stage of liver injury. Liver steatosis is the first structural change in liver secondary to chronic ethanol consumption. There are no clinical symptoms except for hepatomegaly. At the onset of the disease, steatosis is a typical finding, constituting the mildest form of ALD. It is a benign entity and the only clinical symptom in abdominal examination is the finding of an enlarged and tender liver. As damage progresses, the architecture of liver parenchyma changes inducing hepatic fibrosis due to increased collagen deposit. At this stage, patients might suffer decompensated ALD presenting alcoholic hepatitis (AH).

AH is an acute liver inflammation in response to constant injury. The cardinal sign of the disease is the rapid onset of jaundice. Other common signs include fever, ascities and proximal muscle loss. The most common symptoms and clinical signs in alcoholic hepatitis include swollen liver, nausea, vomiting and abdominal pain [31]. The diagnosis is based on a history of heavy alcohol use, jaundice, and the absence of other possible causes of hepatitis [6]. It is usually diagnosed when the liver biopsy indicates inflammatory changes, hepatic degeneration, fibrosis, and other cellular changes. The combination of elevated aspartate aminotransferase level (but <300 IU per milliliter) and a ratio of the aspartate aminotransferase level to the alanine aminotransferase level greater than 2, a total serum bilirubin level above 5 mg per deciliter (86 μmol per liter), an elevated INR, and neutrophilia in a patient with ascites and a history of heavy alcohol use, is indicative of alcoholic hepatitis until proven otherwise. Patients with liver failure resulting from alcoholic liver disease who do not recover within the first 3 months of abstinence are unlikely to survive [6].

When cirrhosis has developed, clinical signs include palmar erythema, contractures in the finger muscles caused by toxic effects of fibrous changes, white nails, “drummer” fingers, liver enlargement or inflammation and fatty infiltration [32]. The clinical presentation is florid and it appears in advanced stages so it is advisable to perform routine liver function tests to patients with a history of ingesting quantities of alcohol capable of increasing health vulnerability, according to WHO recommendations on intake grams of alcohol.

Cirrhosis might be manifested by encephalopathy, which is a clinical manifestation of a severe form of liver disease, as well as malnutrition status and altered liver function tests such as marked prolongation of prothrombin time values, elevation in serum bilirubin above 25 mg/dL, depressed serum albumin, and elevated serum creatinine. Portal hypertension, hepatorenal syndrome, and esophageal varices are the major causes of clinical complications and mortality in ALD [32].

## 5. Diagnostic Tests

### 5.1. Steatosis and Steatohepatitis

Steatosis can be determined with a good level of accuracy by using noninvasive imaging techniques. Currently, there is no available noninvasive diagnostic test to distinguish between steatosis from steatohepatitis or to fibrosis stage [33]. Therefore, liver biopsy remains a useful tool to establish the diagnosis and to exclude other liver disease remaining the only instrument capable to provide prognostic information by staging and grading these diseases. Alcoholic steatohepatitis is a progressive form of alcoholic liver disease and can evolve into cirrhosis. The currently accepted minimum diagnostic criteria for steatohepatitis include steatosis, lobular inflammation, and hepatocellular injury, but fibrosis must be absent. Histologically, hepatocellular injury in fatty liver disease usually occurs in the form of ballooning, but it might also present apoptotic (acidophilic) bodies and lytic necrosis [34]. When there is a good quantity of neutrophils, diagnostic points out to an alcoholic etiology. Another mechanism seems to be sinusoidal collagen formation which results from HSC activation.

### 5.2. Alcoholic Hepatitis

Alcoholic hepatitis is histologically characterized by hepatocellular injury with ballooned hepatocytes. Some of which contain steatosis, whereas others may contain intracellular, amorphous, eosinophilic inclusions called Mallory bodies, which are often surrounded by neutrophils [6].

Hepatic ultrasonography is useful in identifying hepatic abscess, cryptogenic hepatocellular carcinoma, and biliary obstruction, each of which may mimic alcoholic hepatitis. Ultrasonography can also be combined with aspiration of ascites. Doppler flow studies may be useful, since an elevated peak systolic velocity or an increase in the diameter of the hepatic artery may help confirm the diagnosis [3].

### 5.3. Fibrosis and Cirrhosis

**Figure 3.**
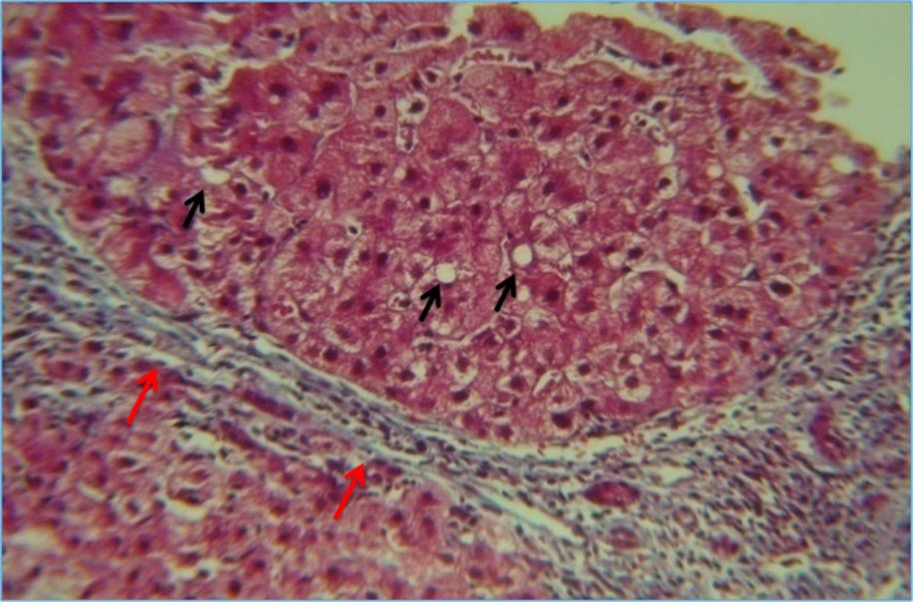
Steatosis (black arrows) with lipidic cytoplasmic inclusions, it is reversible as long as the harmful stimulus is removed. Nodules circumscribed by fibrous tissue with collagen deposition (red arrows).

The characteristic pattern of fibrosis in non-cirrhotic patients with steatohepatitis follows a pericellular/perisinusoidal architecture as the result of collagen deposition in the space of Disse (Figure 3) [35]. Perivenular fibrosis, periportal fibrosis, and cirrhosis, are typical features of alcoholic fibrosis and often coexist with the findings of alcoholic hepatitis. Biomarkers are useful in advanced liver injury but almost useless in the diagnosis of earlier tissular changes.

Emergent new imaging modalities could provide more detailed information about hepatic tissue or even replace biopsy. However, currently there is no method to overpass the specificity of biopsy which, although has disadvantages such as cost, invasiveness, sampling errors, pain, and bleeding risk, provides an accurate staging of liver fibrosis, grade of necroinflammation, steatosis, iron overload, and comorbidities (*i.e.*, autoimmunity) [36].

Recently FibroTest and FibroScan have been used as alternative diagnostic tools. Different studies have demonstrated their efficacy in clinical practice. Nonetheless, liver biopsy is still the gold standard to assess liver fibrosis.

## 6. Complications

High amount of chronic consumption pattern of ethanol increases the risk to develop other health problems. This pattern has a wide variety of adverse effects. It has been already described that ALD involves significant crosstalk among intracellular signaling events in the liver. Overall, inflammatory and innate immune responses in Kupffer cells are activated by elevated gut-derived plasma endotoxin levels [6]. It increases ROS which induces damage and metabolites production, such as acetaldehyde or lipid peroxidation products, that contribute to activation of HSC (the key cell type involved in liver fibrosis) [21]. Complications start when these events diminish the functional hepatocytes. Considering all patients with ALD as a single group, the average 1- and 5-year survival rates are approximately 80 and 50%, respectively [28]. Liver cirrhosis is the most severe form of ALD. Scar tissue replaces normal hepatocytes, disrupting blood flow. When the liver becomes fibrotic and cirrhotic, the number of functional hepatocytes decreases, and the liver loses its capacity to remove toxic substances from the blood [32]. Patients with cirrhosis and superimposed alcoholic hepatitis had a 4-year mortality greater than 60%. The prognosis of individual patients with ALD depends on the degree of pathological injury, the patient's nutritional status, the presence of complications of advanced disease, other comorbid conditions, such as HCV infection, and the patient’s inability to eliminate destructive drinking patterns. Alcoholic cirrhosis also appears to be an independent risk factor for hepatocellular carcinoma [34]. One month mortality in patients with spontaneous hepatic encephalopathy is approximately 50% and 75% in those with hepatorenal syndrome; the median survival of patients with hepatorenal syndrome is less than two weeks. Comparing alcoholic cirrhosis to other etiologies, it is known that alcohol in Japanese society accounts for 13.6 percent of all cases of cirrhosis [11] while Hepatitis B virus (HBV) and HCV infection account for 13.9% and 60.9% of cases respectively. Other causes were primary biliary cirrhosis with 2.4% and autoimmune hepatitis with 1.9% [37].

Alcoholic hepatitis is perhaps the most overwhelming manifestation of alcoholic liver disease. Up to 40% of patients with severe alcoholic hepatitis die within the first 6 months after the onset of the clinical syndrome. It is well known that more severe forms of alcoholic hepatitis are associated with higher mortality rates [38]. Among alcoholic hepatitis signs jaundice and fever may resolve in alcoholic hepatitis within several weeks after discontinuation of alcohol intake, but ascites and hepatic encephalopathy might persist for months to years. Mortality rate in alcoholic hepatitis is about 50% in severe cases. If the heavy drinking pattern continues, 40 percent will develop cirrhosis. When clinical symptoms of encephalopathy appear, ammonia levels in the brain may increase more than twenty fold, this happens in patients with advanced stages of encephalopathy, resulting from chronic liver failure. Ammonia remains the key gut-derived neurotoxin implicated in the pathogenesis of hepatic encephalopathy [39].

## 7. Current Treatment

There is a wide range of therapeutic options for treating ALD and alcohol use disorders. The aim of all therapeutic strategies for ALD is to improve liver function. In alcoholic patients the goal attempts to reduce ethanol intake significantly, looking forward abstinence, thus avoiding further hepatic damage.

### 7.1. Alcoholic Liver Disease

Prognosis of individual patients with ALD depends on the degree of histological injury, patient’s nutritional status, presence of complications of advanced disease, other comorbid conditions such as HCV infection, and the patient's ability to eradicate destructive drinking patterns. The cornerstone in ALD therapy is a daily life style modification, including drinking cessation and treatment of decompensation, if appropriate. Abstinence from alcohol is vital in order to prevent further ongoing liver injury, fibrosis, and possibly hepatocellular carcinoma [33].

#### 7.1.1. Nutrition

Several studies support the notion that nutritional status is a determinant factor in the outcome after any therapy. Nutritional intervention has shown to play a positive role on both, in-patient and out-patient basis. Data has described patients with alcoholic liver injury who voluntarily consumed >3,000 kcal/day had virtually no mortality, whereas those consuming <1,000 kcal/day had a mortality greater than 80% at 6-month. Moreover, the undernourishment degree in cirrhosis was correlated with the development of severe complications such as encephalopathy, ascites and hepatorenal syndrome. Jonas *et al*. found that alcoholic fatty liver decreased significantly in rats with fatty acids supplemented diet, this was observed when oxidative/nitrosative levels were normalized in rats fed with alcohol-fatty acids-supplementes diet [40]. The molecular mechanism of DHA/AA, (decosahexaenoic acid/arachidonic acid) essential fatty acids, against liver steatosis is poorly understood. However, it has been observed that steatosis increases in diets with greater amounts of polyunsaturated fatty acids. This fact indicates a sharpened threshold between therapeutic and harmful effects [5]. In cirrhotic patients it has been demonstrated that a nutritious diet improved the 5-year outcome compared with patients consuming an inadequate diet.

#### 7.1.2. Pharmacotherapy

The progression of ALD continues according to the amount of alcohol consumed. Complications appear when there is a considerable liver fibrosis tissue, few functional hepatocytes, and when chronic inflammation affects the structure and function of the liver. When progression renders alcoholic hepatitis, corticosteroids are widely used in selected patients and treatment with pentoxifylline appears to be a promising anti-inflammatory therapy. Recent studies have indicated anti-TNFα therapy, at least for alcoholic hepatitis, although further clinical trials must be designed due to the disparity in the results of some research data. However antioxidant therapy, alone or in combination with corticosteroids, does not improve 6-month survival in severe alcoholic hepatitis [30]. Our own studies with pirfenidone have suggested an important anti-inflammatory effect with ALD patients (Armendariz-Borunda, *et al*., unpublished results).

The treatment of alcoholic hepatitis is largely symptomatic, emphasizing alcohol abstinence and meticulous attention to nutritional status. Specific therapies such as corticosteroids and pentoxyfiline need to be considered as they may reduce mortality in those patients who are at high risk of dying [41].

#### 7.1.3. About transplant

Currently, the two main indications for liver transplant are: ALD and liver failure caused by HCV infection both in Europe and North America. Alcoholic hepatitis remains a controversial indication for liver transplantation; currently the procedure is not performed in this these patients owing to the fact that they continue consuming alcoholic beverages. Furthermore, it is required a period of abstinence as a rule before liver transplantation in many clinics. At the end of this period, liver functionality improves significantly in most of the cases so the process is not performed. Although it has been reported that transplant outcome in ALD and other entities, such as chronic HVC infection, is very similar, in the daily practice many transplants are interrupted for the reasons previously described [42].

### 7.2. Alcohol Use Disorders

Alcoholism is a multifactorial entity; hence the treatment must be addressed with different strategies related to AD. Alcohol use disorders are treatable disorders when efficacious medicines are added to enhance the effects of psychosocial treatment. Due to personal, social and health impact of alcohol use disorders, therapy must be guided towards a complete management including pharmacotherapy, behavioral interventions and medical board. For this reason, different strategies have been developed. Although abstinence remains the ultimate goal in treating alcohol-dependent individuals, reducing the frequency of heavy drinking has the major impact on decreasing alcohol-related consequences and improving quality of life [43].

#### 7.2.1. Pharmacotherapy

The most promising compounds appear to be those that modulate the function of opioids, glutamate with or without gamma-aminobutyric acid (GABA), and serotonin (5-HT) [43]. The most used and approved pharmacotherapy by the Food and Drug Administration of the United States is naltrexone, acamprosate and disulfiram.

Naltrexone is an opioid antagonist which is prescribed for alcoholism treatment [44]. Oral naltrexone and naltrexone depot formulations have generally demonstrated efficacy in treating AD, but with small effect. Treatment with naltrexone works for some individuals, but not for everyone. It has been proved a functional polymorphism of the μ-opioid receptor (OPRM1) gene that might predict naltrexone response. Anton *et al*. demonstrated the relationship between the functionally significant *OPRM1* Asp40 allele and the response to naltrexone in alcoholic patients. Carriers of the Asp40 allele had an increased percentage of abstinent days (*P* = 07) and a decreased percentage of heavy drinking days (*P* = 0.04). The homozygous Asn40/Asn40 genotype showed no medication differences [45].

GABA and glutamate systems are particularly sensitive to alcohol effects, showing adaptation during the course of long-term alcohol exposure [46], for this reason receptor agonists have been studied as therapy targets for alcoholism. The limitations using these drugs lay on the changes in the gabaergic system, more specifically in the GABA A receptors in rats after chronic alcohol feeding. Among the new promising drugs for alcoholism we point to topiramate. It facilitates inhibitory GABA A-mediated currents at non-benzodiazepine sites on the GABA A receptor [43].

Baclofen is a GABA B receptor agonist which has demonstrated good outcome in small clinical trials, however further research is needed. Although neither topiramate nor baclofen have been approved by the FDA, these are among the most promising drugs, with a medium effect size in clinical trials. Furthermore serotonergic agents such as selective serotonin reuptake inhibitors and the serotonin-3 receptor antagonist, ondansetron, appear to be efficacious only among certain genetic subtypes of alcoholics [43]. Biological pathway of GABA is probably involved in AD, a key area for proteomics which consists in a biomarker discovery that could correlate protein expression with the treatment outcome or global prognosis of the disease [47].

Duloxetine is a selective serotonin/norepineprhine reuptake inhibitor (SSNRI) and has been proposed as a new therapy for alcohol use disorders. The results in rats treated with duloxetine showed robust reductions in alcohol consumption. *Post-hoc* analysis indicated that all doses of duloxetine significantly suppressed binge-like alcohol intake (*P* < 0.05) in all cases [44]. Nonetheless relapse in drinking and craving in the postdetoxification period is noticed, more than if treated with gabapentin.

Disulfiram inhibits aldehyde dehydrogenase and prevents the metabolism of alcohol’s primary metabolite, acetaldehyde. The accumulation of acetaldehyde in the blood causes unpleasant effects. This drug has been accepted by the FDA since 1940 for treating AD, although craving for alcohol is not reduced and it has been related to toxicity and severe adverse effects. However it is still widely used for the treatment of alcoholism.

Johnson *et al*. report that acamprosate has demonstrated efficacy for treating AD in European trials, but not so in the United States, with up to 70% of patients resuming drinking within one year. The use of acamprosate thus presents inconclusive results. It has been approved by the FDA, however it hasn’t shown positive results when comparing its effects with placebo. Unlike in the U.S., European studies show some efficacy in the use of acamprosate [43].

In the COMBINE study, data show that patients receiving treatment with naltrexone, combined behavioral intervention or both, fared better on drinking outcomes, whereas acamprosate showed no evidence of efficacy, with or without combined behavioral intervention [48]. New pharmacological agents that inhibit 5-HT reuptake from the synapse, reduce the voluntary consumption of ethanol.

#### 7.2.2. Alcoholics anonymous

Alcoholics Anonymous (AA) is the most commonly used program for substance abuse recovery and one of the few models to demonstrate positive abstinence outcomes. Social support is a mechanism in the effectiveness of AA in promoting a sober lifestyle and worldwide membership is estimated at over 2,000,000 in 150 countries. Some studies reported that the participation of AA was linked to positive drinking outcomes and modestly related to better psychological health, social functioning, employment situation, and legal situation [49].

## 8. Conclusions

Ethanol consumption is widely accepted worldwide and it is sometimes traduced into substance abuse. It causes global public health problems, covering social aspects as well as psychological, economical and family ones, among others.

When alcohol consumption affects an individual’s health, molecular mechanisms inducing liver damage begin to occur. These processes have been described broadly and therapies developed for these diseases are focused on avoiding alcohol abuse and improvement of liver function, once a diagnosis of ALD has been made.

Nevertheless, there are genetic factors that could predispose to the development of alcoholism or ALD. The identification of certain polymorphisms associated to these entities has been possible. Currently, there is a lengthy list of treatment strategies. Both conditions have multifactorial origin, so each factor involved is a target for the development of new therapies. New drug research is needed since currently available treatments are not as effective as expected, for after a year of alcohol consumption cessation, pathological drinking patterns tend to reappear.

## Figures and Tables

**Table 1.** Causes of death related to alcohol abuse.

1	Mouth and oropharynx cancer
**2**	Alcohol use disorders
**3**	Ischemic heart disease
**4**	Liver cirrhosis
**5**	Road traffic accidents
**6**	Poisonings
**7**	Falls
**8**	Intentional injuries
